# Antimicrobial capacity of Leucocyte-and Platelet Rich Fibrin against periodontal pathogens

**DOI:** 10.1038/s41598-019-44755-6

**Published:** 2019-06-03

**Authors:** Ana B. Castro, Esteban R. Herrero, Vera Slomka, Nelson Pinto, Wim Teughels, Marc Quirynen

**Affiliations:** 1 Department of Oral Health Sciences, Periodontology, KU Leuven & Dentistry, University Hospitals Leuven, Leuven, Belgium; 2 Faculty of Dentistry, Postgraduate Implant Program, University of the Andes, Santiago, Chile

**Keywords:** Applied microbiology, Periodontitis

## Abstract

Various studies have described the biological properties of the Leucocyte- and Platelet Rich Fibrin (L-PRF) such as the antimicrobial effect against wound bacteria, but less is known about the effect against periodontal pathogens. The aim of this study was to evaluate the antibacterial properties of the L-PRF membrane and L-PRF exudate against the main periopathogens cultured on agar plates and in planktonic solution. This study demonstrated the antibacterial effect of the L-PRF membrane against *P*. *intermedia*, *F*. *nucleatum*, and *A*. *actinomycetemcomitans*, but especially against *P*. *gingivalis*. The L-PRF exudate also showed a strong inhibition against *P*. *gingivalis* on agar plates. No inhibition could be observed for the other bacterial strains. Moreover, L-PRF exudate decreased the number of viable *P*.*gingivalis* in a planktonic solution in a dose-dependent way. However, *A*. *actinomycetemcomitans* showed an increased growth in planktonic solution when in contact with the L-PRF exudate.

## Introduction

The classic experimental gingivitis studies in the 60 s^1,2^ demonstrated the direct relation between the accumulation of dental plaque and gingival inflammation. Dental plaque or dental biofilms are defined as a matrix-embedded microbial population, adherent to each other and/or to surfaces or interfaces^3^. Studies on biofilm development in deep periodontal pockets showed that the deepest sites are colonized predominantly by motile species (e.g. spirochetes) and gram-negative bacteria, located adjacent to the epithelial lining of the pocket. Some of these bacteria are part of the red complex (*Porphyromonas gingivalis*, *Treponema denticola*, *Tannerella forsythia)* and the orange complex (e.g. *Fusobacterium nucleatum)*^4^. On the other hand, gram-positive rods and cocci are more dominant in shallow sites, forming a firmly adherent band of microorganisms on the enamel or root surface^4–6^. One of the most solid associations between a periodontal pathogen and destructive periodontal disease is provided by *Aggregatibacter actinomycetemcomitans* JP2, a potential etiologic agent of localized aggressive periodontitis^7–9^.

Traditionally, the goal of non-surgical periodontal therapy is to eliminate the microbial and/or inflammatory etiology and to reduce the initial pockets to ≤5 mm. Antimicrobial agents are often used as adjuncts to initial therapy^10^. If the desired pocket depths are not achieved, surgical treatment is required^11,12^.

Recently, new tissue-engineering techniques have been proposed for regenerative procedures after non-surgical periodontal therapy^13^ or for bone augmentation^14,15^. Leucocyte- and platelet-rich fibrin (L-PRF), a second-generation platelet concentrate, was introduced as an autologous biomaterial that serves as a scaffold for regenerating cells. L-PRF is prepared from the patient’s own blood, without adding any additives, and concentrates >90% of the platelets and >75% of the leucocytes from the initial blood composition^16^. It offers a continuous release of growth factors and other bioactive substances that stimulate and protect the surgical site^17,18^. Different forms of L-PRF can be prepared, the membrane most commonly used.

Two recent systematic reviews^19,20^ have shown the various applications of L-PRF, concluding that favourable effects on hard and soft tissue healing and a decrease of postoperative discomfort could be obtained when L-PRF was used. Several studies have described other biological properties of L-PRF such as the antimicrobial effect against wound bacteria^21,22^. Given the cellular composition of L-PRF^16^ and its bioactive nature^17^, the aim of this study was to evaluate the antimicrobial capacity of an L-PRF membrane and L-PRF exudate against key periodontal pathogens (*Porphyromonas gingivalis*, *Prevotella intermedia*, *Fusobacterium nucleatum*, and *Aggregatibacter actinomycetemcomitans*). The null-hypothesis was postulated as (1) an L-PRF membrane and its exudate do not inhibit the four tested bacterial strains when applied directly on a cultured Brain Heart Infusion (BHI) agar plate, and (2), L-PRF exudate does not inhibit bacterial growth of the tested bacterial strains in planktonic form.

## Results

### Demographic data

Nine systemically healthy adult volunteers (6 women, 3 men) participated in this study. Their mean age was 37.7 ± 15.3 years (range 25–60 years). No complications were reported during blood collection.

### Agar plate test

#### L-PRF membrane

The mean area of inhibition was 11.8 ± 5.0 mm^2^, 2.7 ± 5.2 mm^2^, 2.6 ± 3.0 mm^2^, and 0.6 ± 1.7 mm^2^ for *P*. *gingivalis*, *P*. *intermedia*, *F*. *nucleatum*, and *A*. *actinomycetemcomitans*, respectively (Table 1). More inhibition was found for *P*. *gingivalis* when compared to *P*. *intermedia* (p < 0.05), *F*. *nucleatum* (p < 0.05), and *A*. *actinomycetemcomitans* (p < 0.05).

**Table 1:** Dimensions of L-PRF membranes at baseline and after 72 h of incubation as well as the difference between both observations (▲), and bacteria-free area (mm^2^) for nine participants (mean and standard deviation). *p < 0.05.

L-PRF membrane		P. gingivalis		P.intermedia		F. nucleatum		A. actinomycetemcomitans	
		mean	sd	mean	sd	mean	sd	mean	sd
**Length and Width (mm)**									
Baseline	length	39.4	3.3	35.9	3.5	35.8	4.7	35.1	7.0
	width	10.5	1.2	11.3	1.0	11.1	2.3	10.4	1.3
72 h	length	39.0	3.3	34.2	3.2	35.1	4.6	34.3	6.4
	width	8.8	4.1	9.7	3.8	9.5	3.9	8.9	3.8
▲	length mm	0.4	0.6	1.6	0.2	0.7	0.7	0.8	0.8
	width mm	0.6	0.5	0.4	0.5	0.3	0.2	0.4	0.3
	length %	1.1	1.6	4.4	1.5	2.1	2.0	2.1	1.7
	width %	6.7	4.9	3.7	2.9	5.5	7.6	4.1	3.5
**Surface** (**mm**^2^)									
Baseline	membrane surface	37.0	4.6	33.3	6.0	35.4	5.1	32.2	7.9
72 h	membrane surface	34.4	4.4	31.1	11.1	33.6	5.0	30.5	7.0
▲	shrinkage mm^2^	2.7	1.1	2.1	1.1	1.9	0.9	1.7	2.0
	shrinkage %	7.2	2.9	5.5	3.9	5.3	2.5	4.8	5.1
**Area of bacterial growth inhibition (mm** ^2^ **)**									
72 h	full area	11.8*	5.0	2.7	5.2	2.6	3.0	0.6	1.8
	area without shrinkage	9.1*	3.2	0.5	1.3	0.5	0.9	0.2	0.7

Over the 72-hour time incubation period, a shrinkage of the membranes was observed. The mean shrinkage of the L-PRF membrane was 5.7 ± 3.7% after 72 h of incubation at 37 °C in both aerobic and anaerobic conditions. The mean reduction in length and width was 1.0 ± 0.5 mm and 0.5 ± 0.1 mm, respectively. None of these changes (Table 1) were statistically significant (p > 0.05).

However, since membrane shrinkage could have influenced the mean area of inhibition, it was subtracted from the area of inhibition. After subtracting membrane shrinkage, *P*. *gingivalis* showed an area of inhibition of 9.1 ± 3.2 mm^2^ (p < 0.05). For *P*. *intermedia*, *F*. *nucleatum* and *A*. *actinomycetemcomitans* no statistically significant inhibition could be measured (p > 0.05).

#### L-PRF exudate

For the L-PRF exudate, only inhibition against *P*. *gingivalis* could be observed, with a mean area of inhibition of 17 ± 2.6 mm2 (Table 2). For the positive control (CHX 0.12%), the mean area of inhibition was statistically significantly larger (48.8 ± 4.2 mm2, p < 0.005).

**Table 2:** Mean bacteria-free area (mm^2^) and standard deviation for the L-PRF exudate and chlorhexidine 0.12% (positive control). Data for nine participants. *p < 0.05.

L-PRF exudate (mm^2^)		P. gingivalis		P. intermedia		F. nucleatum		A. actinomycetemcomitans	
		mean	sd	mean	sd	mean	sd	mean	sd
72 h	L-PRF exudate	17.5*	2.0	0.0	0.0	0.0	0.0	0.0	0.0
	chlorhexidine 0.12%	48.0*	4.2	79.4*	7.0	28.8*	4.0	27.4*	3.0

### Planktonic bacteria

#### Vitality qPCR

**Figure 1:**
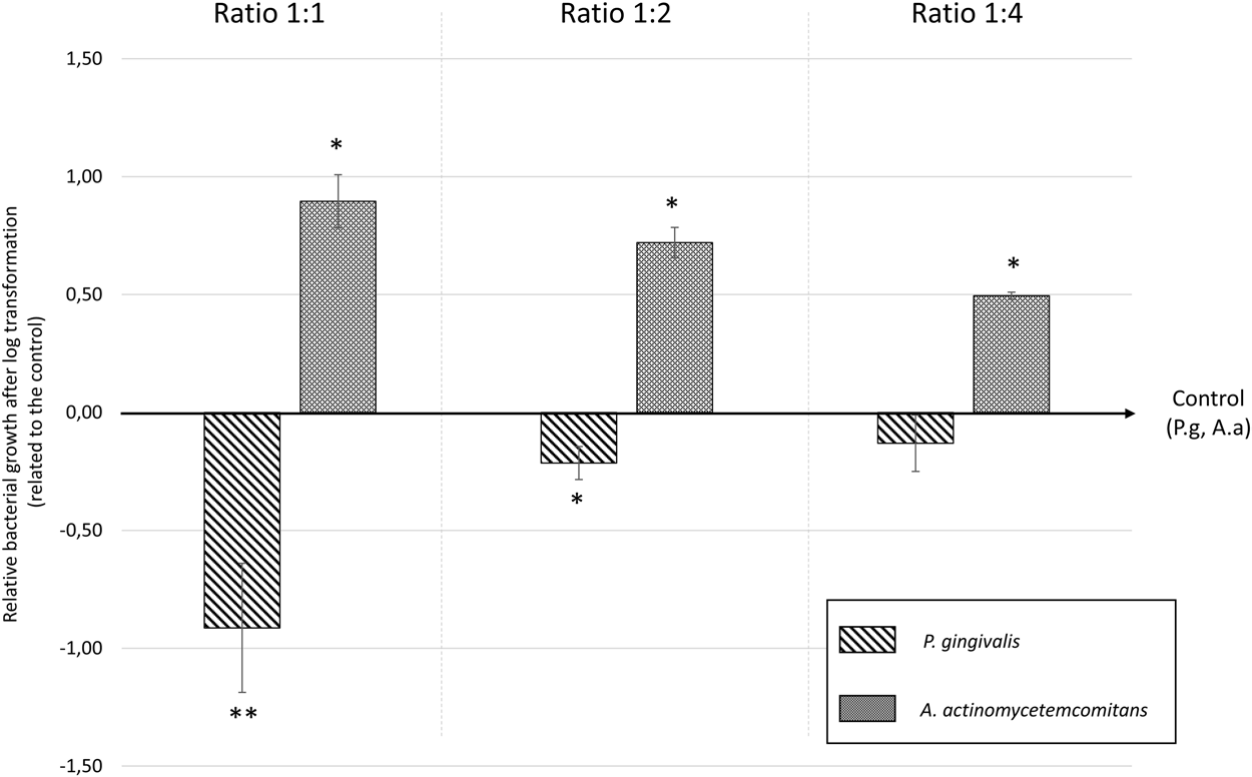
Effect of L-PRF exudate (1:1, 1:2, and 1:4 ratios) on *P*. *gingivalis* and *A*. *actinomycetemcomitans* in planktonic form. Results of the vitality qPCR shown after log transformation related to the control (P.g and A.a, respectively). Positive values represent bacterial growth. Negative values indicate bacterial inhibition. **p < 0.001; *p < 0.05.

*P*. *gingivalis* and *A*. *actinomycetemcomitans* were considered for this analysis since they showed the highest and the lowest amount of inhibition induced by the membranes, respectively. The vitality qPCR analysis showed a mean reduction of 0.9 log (86%, p < 0.001), 0.2 log (38%, p < 0.05), and 0.1 log (24%, p > 0.05) in the growth of *P*. *gingivalis* when exposed to the L-PRF exudate with the ratios 1:1, 1:2, and 1:4, respectively. For the *A*. *actinomycetemcomitans*, a mean increase of 0.9 log (p < 0.05), 0.7 log (p < 0.05), and 0.5 log (p < 0.05) was observed for the respective ratios (Fig. 1).

#### Colonies counting (CFU/ml)

For *P*. *gingivalis*, the standard culture test showed a mean of 2.4 × 10^12^ CFU/ml in the control condition and of 9.4 × 10^9^, 3.8 × 10^10^, and 2.0 × 10^11^ for the ratios 1:1, 1:2, and 1:4, respectively. For *A*. *actinomycetemcomitans*, the mean CFU/ml for the control was 3.1 × 10^7^, whereas the counting was 3.0 × 10^11^, 1.8 × 10^11^, and 5.5 × 10^10^ for the respective ratios 1:1, 1:2, and 1:4 (Table 3).

**Table 3:** Mean (±standard deviation, SD) qPCR and CFU counting (log values) for P. gingivalis and *A*. *actinomycetemcomitans*.

		Vitality qPCR (Log_10_ Geq/ml)		Culturing (Log_10_ CFU/ml)	
	Groups	mean	*sd*	Mean	*sd*
*P*. *g*	Control	9.6	*0*.*08*	12.4	*0*.*6*
	1:1	8.6	*0*.*4*	9.7	*0*.*4*
	1:2	9.4	*0*.*2*	10.4	*0*.*4*
	1:4	9.5	*0*.*2*	11.0	*0*.*5*
***A***. ***a***	Control	10.4	*0*.*2*	7.5	*0*.*2*
	1:1	11.3	*0*.*3*	11.2	*0*.*6*
	1:2	11.1	*0*.*3*	11.2	*0*.*3*
	1:4	10.9	*0*.*2*	10.7	*0*.*3*

Control: 150 µl bacteria + 150 µl saline. Ratio 1:1: 150 µl bacteria + 150 µl L-PRF exudate, ratio 1:2: 150 µl bacteria + 75 µl L-PRF exudate + 75 µl saline, and ratio 1:4: 150 µl bacteria + 37.5 µl L-PRF exudate + 112.5 µl saline. Data for nine volunteers.

#### Gram negative staining

**Figure 2:**
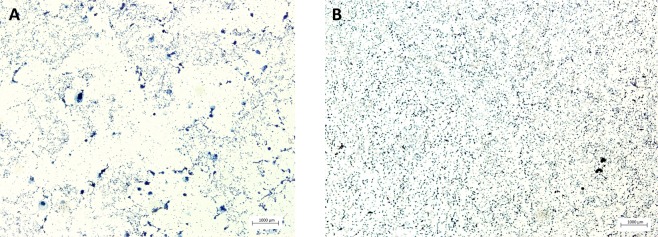
Gram negative staining of *A*. *actinomycetemcomitans*. (**A**) Control, bacteria in aggregated clusters; (**B**) ratio 1:1, bacteria in separate colonies.

In order to find an explanation for the differences observed between the vitality qPCR data and the culture data for *A*. *actinomycetemcomitans*, gram staining was performed. As shown in Fig. 2, in the control group (Fig. 2A) *A*. *actinomycetemcomitans* formed aggregated clusters known as auto-aggregation. In the ratio 1:1, the bacteria appeared looser than those in the control group and no auto-aggregation was observed (Fig. 2B). Since clusters of bacteria were counted as one colony in the culturing method, and, all bacteria were counted as independent bacteria in the qPCR analysis, the discrepancy between culturing and vitality qPCR could be explained via inhibition of auto-aggregation.

## Discussion

The results in this study demonstrated antimicrobial activity of the L-PRF membrane against *P*. *gingivalis*. No statistically significant inhibition could be observed for *P*. *intermedia*, *F*. *nucleatum*, and *A*. *actinomycetemcomitans*. The L-PRF exudate also showed a clear inhibition against *P*. *gingivalis* on agar plates. In planktonic form, a dose-dependent inhibition against *P*. *gingivalis* was observed for the L-PRF exudate, whereas for *A*. *actinomycetemcomitans* an increase in bacterial growth was detected.

Several studies showed an antimicrobial effect of platelet concentrates against wound bacteria. Burnouf and co-workers^21^ described bacterial inhibition by platelet-rich plasma (PRP) against *Pseudomonas aeruginosa*, *Staphylococcus aureus*, and *Escherichia coli* in planktonic form, after 3 h of aerobic incubation. Similar results were reported by Edelblute and co-workers^22^ on using a clotted form of platelet-rich plasma (PRP), after having added bovine thrombin. They concluded that the human platelet gel supernatant inactivated opportunistic pathogens on the skin, such as *S*. *aureus* and *Acinetobacter baumannii*, but not *P*. *aeruginosa*. However, controversial results were shown by Chen and co-workers^23^ stating that PRP had an antibacterial effect against *S*. *aureus* but not against *E*. *coli* or against *P*. *aeruginosa* in patients with diabetes.

Only few articles examined oral microorganisms. For instance, *Enterococcus faecalis* and *Candida albicans* isolated from the oral cavity were inhibited by pure-platelet-rich plasma (P-PRP), a type of PRP without leucocytes^24,25^. Yang and co-workers^26^ showed inhibition on *P*. *gingivalis* and *A*. *actinomycetemcomitans* by PRP whereas no inhibition could be observed for *F*. *nucleatum*. In this study, the effect of PRP and PRF was compared, concluding that PRP showed superior activity. It should be noted that PRF was prepared by adding calcium chloride to PRP in order to activate the platelets and convert fibrinogen into fibrin, which is not in line with the protocol to prepare PRF. Moreover, the authors affirmed that in their study PRF had neither platelets nor leucocytes. However, by definition, platelet concentrates are blood-derived products with an increased concentration in platelets, with or without the presence of leucocytes^25,27^. Therefore, these results must be interpreted with caution. More recently, Kour and co-workers (2018)^28^ compared the antibacterial capacity of PRP, PRF, and injectable-PRF (I-PRF) on *P*. *gingivalis* and *A*. *actinomycetemcomitans*. They concluded that all three platelet concentrates showed some antibacterial activity against both bacteria. However, PRP and I-PRF presented significantly greater inhibition against *P*. *gingivalis* compared to PRF. In the present study, limited inhibition could be observed on A. *actinomycetemcomitans*, which differs from their results. The bacterial strain used and the fact that they incubated A. *actinomycetemcomitans* anaerobically might have influenced the effect of PRF.

*P*. *gingivalis* was the most inhibited bacteria strain in this study. However, there is no evidence in the literature showing a direct effect of blood components on *P*. *gingivalis*. Proteinase inhibitors constitute 10% of the protein content of human plasma and they can affect the gingipain activity, known as the primary virulence factor of those bacteria. These proteinases also play an important role in the survival of the bacterium within host cells, due to their implication in the cellular invasion and in overcoming the protective defence mechanisms of epithelial cells^29,30^. An example of proteinase inhibitors is the human alpha-2-macroglobulin, a large plasma protein found in blood, that inhibited RgpA and RgpB, but not Kgp in an *in-vitro* study^31^. The alpha-granules from the platelets are also an important intracellular storage of biologically active proteins^32,33^. Seven antimicrobial peptides have been identified from human platelets, suggesting a direct antimicrobial role^34^. However, the direct relation between those peptides and the antimicrobial capacity of the L-PRF has not been proved yet. For the L-PRF membrane, two theories could be advocated: (1) the living cells inside the fibrin matrix constantly released antimicrobial peptides against the targeted bacteria, or (2) the antimicrobial peptides were entrapped in the fibrin matrix and they were gently released as the fibrin matrix was being disintegrated. Concerning the L-PRF exudate, no specific pathways are known between blood-derived products and *P*. *gingivalis*. Therefore, further research should investigate this association.

Two effects on *A*. *actinomycetemcomitans* could be observed in this present study. First, there was an effect on the auto-aggregation of these bacteria. One possible explanation is that the L-PRF exudate might impair the aggregation of those bacteria^35^. Given that most bacteria exist in environments with fluctuating conditions (e.g. shear forces, nutrient availability or physiological conditions), the bacteria within co-aggregated communities will survive and proliferate under conditions that reduce the prevalence of single non-aggregated cells. For these reasons, co-aggregation processes are likely to have an important ecological role in the development and maintenance of multiple-species biofilm^36,37^. Regarding the second effect, bacterial growth stimulation of *A*. *actinomycetemcomitans* was detected when it was in contact with the L-PRF exudate. Fresh human serum is known for enhancing leukotoxic activity of these bacteria^38,39^. Johansson and co-workers^40^ indicated that the increased leukotoxicity of A. actinomycetemcomitans observed in the presence of human serum is caused by the serum protease inhibitors, which counteract the proteolytic degradation of the leukotoxin.

To the best of our knowledge, this was the first time that the shrinkage of the L-PRF membrane was considered, in surface as well as in length and width. A mean surface shrinkage of 5% *in vitro* after 3 days was reported. These results cannot be straightforwardly extrapolated to the clinical situation. However, they provide insight into the dimensional changes that might occur when used in the oral cavity. It must be noted that the shrinkage of the L-PRF membrane probably occurred progressively. The area of shrinkage was thus gradually exposed, but not colonised by the surrounding bacteria. The explanation for the absence of microbial growth in the area that became exposed after membrane shrinkage is more complex, including factors such as antimicrobial activity at distance, antimicrobial activity due to direct contact or growth inhibition due to mechanical coverage.

Within the limitations of this study, it can be concluded that an L-PRF membrane has antimicrobial properties against *P*. *gingivalis*, whereas the inhibition against *P*. *intermedia*, *F*. *nucleatum*, and *A*. *actinomycetemcomitans* was not statistically significant on agar plates. The L-PRF exudate showed a strong inhibition against *P*. *gingivalis* on agar plates, but no inhibition could be observed for the rest of the bacterial strains. Therefore, we cannot reject the first null-hypothesis. For the L-PRF exudate, the second hypothesis cannot be rejected because *A*. *actinomycetemcomitans* even showed increased growth. However, the L-PRF exudate has an antimicrobial effect against *P*. *gingivalis* in a dose-dependent way.

## Methods

### Bacterial strains

All bacterial species (*P*. *gingivalis* ATCC 33277, *P*. *intermedia* ATCC 25611, *F*. *nucleatum* ATCC 20482, *A*. *actinomycetemcomitans* ATCC 43718) were maintained on blood agar (Oxoid, Basingstoke, UK) supplemented with 5 mg/ml hemin (Sigma, St. Louis, USA), 1 mg/ml menadione (Calbiochem-Novabiochem, La Jolla, CA, USA), and 5% sterile horse blood (E&O Laboratories, Bonnybridge, Scotland). Overnight liquid cultures were prepared in BHI broth (Difco, Detroit, MI, USA). The bacteria were cultured under anaerobic conditions (80% N_2_, 10% H_2_ and 10% CO_2_) for *P*. *gingivalis*, *P*. *intermedia*, and *F*. *nucleatum* or aerobic conditions (5% CO_2_) for *A*. *actinomycetemcomitans*.

### Leucocyte- and Platelet-Rich Fibrin (L-PRF) preparation

The participants in this study (n = 9) were systemically healthy, non-smokers, without a history of periodontal disease and who had not taken any antibiotics for at least 6 months before the study. Four blood samples were collected from each volunteer (n = 9) in a 9 mL glass-coated plastic tube and immediately centrifuged at 408 *g* for 12 minutes (Intraspin™, Intra-Lock, Boca Raton, FL, USA). The L-PRF clot was compressed and transformed into a standardized membrane of 1 mm in thickness (Xpression® box, Intra-Lock, Boca Raton, FL, USA). During this process, the released exudate was also kept for further use at −80 °C. Due to the low cellular content of L-PRF exudate (<2.5% of platelets and <0.9% of leucocytes for the initial blood composition)^41^, we could freeze it without damaging the possible active molecules.

### Agar plate test

#### L-PRF membrane

BHI agar plates (Difco, Sparks, MD, USA) were seeded with an overnight culture 2 h before the application of an L-PRF membrane and incubated in anaerobic (80% N_2_, 10% H_2_ and 10% CO_2_) or aerobic (5% CO_2_) conditions, depending on the bacteria. The L-PRF membranes on the surface of a BHI agar plate were in direct contact with *P*. *gingivalis*, *P*. *intermedia*, *F*. *nucleatum* or *A*. *actinomycetemcomitans*. The incubation time was 72 h.

**Figure 3:**
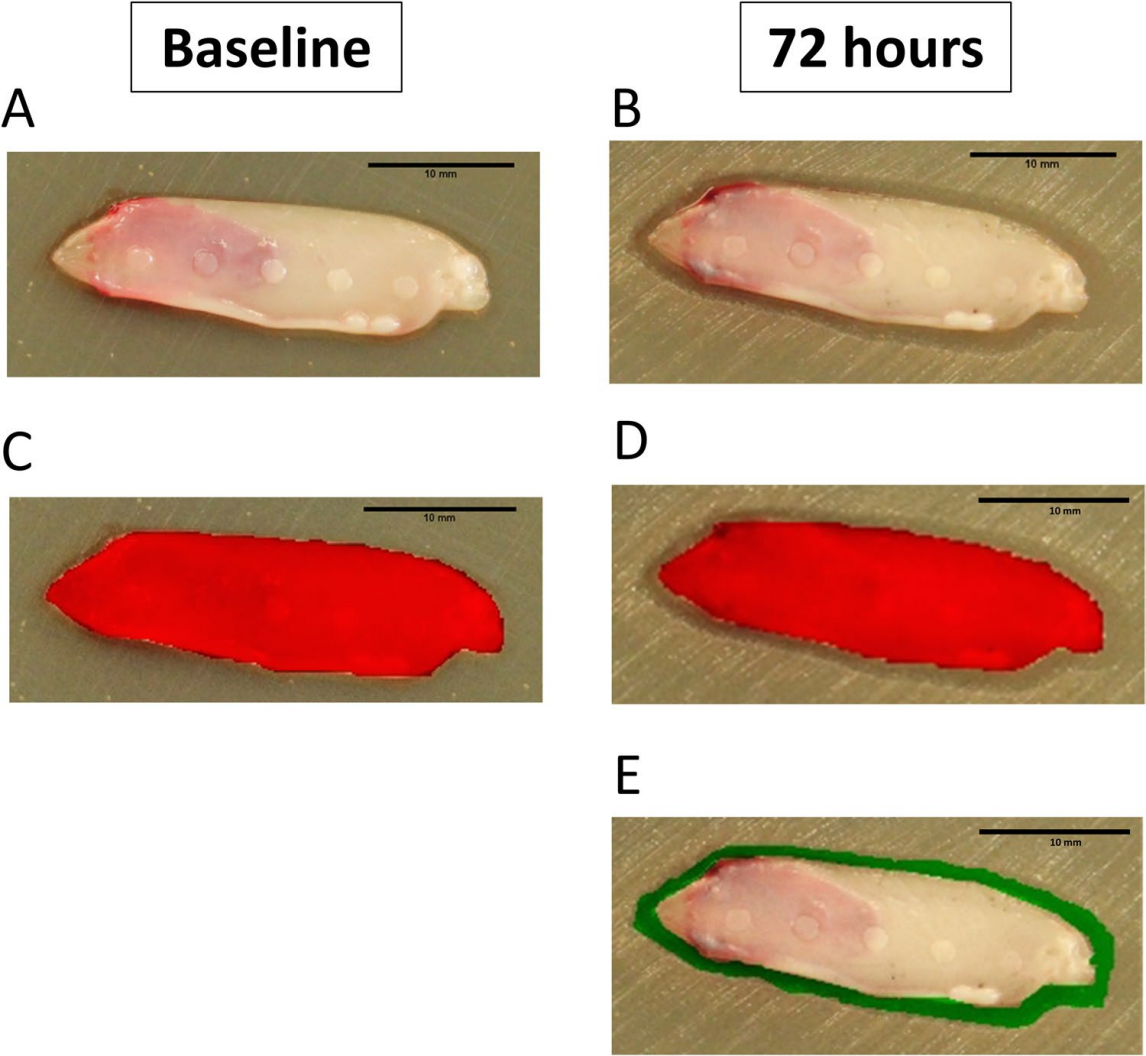
(**A**) L-PRF membrane on BHI agar plate previously inoculated with an overnight culture of *P*. *gingivalis*. (**B**) Calculation of initial membrane surface area using PictZar® software (red area). (**C**) After 72 hours of anaerobic incubation, bacterial growth became visible (white colonies). (**D**) Calculation of the membrane’s new surface area (red area), in order to detect membrane shrinkage. (**E**) Calculation of area without bacterial growth (green area).

Standardized pictures (constant distance between agar plate and camera) were taken at baseline and after 72 h. In order to verify the shrinkage, the difference in surface area of the membrane between pictures at baseline and those after 72 h was computed with PictZar® Pro 7.1 (Digital Planimetry Software). Each image was calibrated based on the ruler that was attached to the agar plate. First, the area of the L-PRF membrane at baseline was coloured (red) and its area was calculated. The same was done for the L-PRF membrane after 72 h of incubation. The area free of bacterial growth around each membrane was coloured (green) and calculated. To evaluate the shrinkage, the area of the L-PRF membrane at baseline was subtracted from the area after 72 h (Fig. 3).

The length and width of each membrane were measured at baseline and after 72 h with the software ImageJ® (Image Processing and Analysis in Java, 1.8.0_77). All images were individually calibrated based on the ruler that was fixed to each agar plate. A horizontal (length) and a vertical (width) line were drawn from the middle point of the membrane with an angle of 90°.

#### L-PRF exudate

Twenty-five microliters of L-PRF exudate, 25 µl of pure chlorhexidine (CHX) 0.12% (positive control) and 25 µl of BHI broth (negative control) were applied directly to the surface of each BHI agar plate, prepared and inoculated in the same way as in the previous experiment. The incubation time was 72 h.

Standardized pictures were taken at baseline and after 72 h. The area free of bacterial growth around each drop of L-PRF exudate was measured with the software PictZar® Pro 7.1.

### L-PRF exudate dilution test

If inhibition with L-PRF exudate was detected, bacteria were cultured in BHI broth and optical densities were adjusted using spectrophotometry (OD600, GeneQuant Spectrophotometer, Buckinghamshire, UK) to an OD of 0.5–0.7 (OD_600_ = 0.5 ≈ 1 × 10^8^ colony forming unit/ml (CFU/ml). Three dilutions of L-PRF exudate were prepared in a 96-well plate, for a total volume of 300 µl/well: ratio 1:1 (150 µl of bacteria + 150 µl of L-PRF exudate), ratio 1:2 (150 µl of bacteria + 75 µl of L-PRF exudate + 75 µl of saline), and ratio 1:4 (150 µl of bacteria + 37.5 µl of L-PRF exudate + 112.5 µl of saline). The positive control consisted of 150 µl of bacteria + 150 µl of saline. The incubation time was 24 h. One bacterial strain that was not inhibited during the agar plate test was also cultured.

After 24 h, 90 µl of the mixtures mentioned above was taken for vitality quantitative PCR (qPCR). Another 50 µl was used for microbial culturing and colony counting (CFU/ml). This experiment was performed in triplicate.

#### Vitality qPCR

The vitality-DNA extraction was performed with a QIAamp DNA Mini kit (Qiagen, Hilden, Germany)^42^. Briefly, 90 μl aliquots of the samples were immediately incubated with 10 μl of propidium monoazide (PMA) (Biotium, Hayward, CA, USA) at a final concentration of 100 μg/ml^42^. Samples were incubated in the dark for 5 min, following photo-induced cross-linking of PMA by 10-min light exposure using a 400 W (500 lm) light source, placed 20 cm above the sample, while samples were kept on ice. The PMA treated bacteria were pelleted by centrifugation at 20,000 × g for 10 min and DNA extraction was performed using the QIAamp DNA Mini kit following the manufacturer’s instruction, extending the incubation step of the bacteria in the lytic enzyme solution at 37 °C to 2 h.

A qPCR assay was performed with a CFX96 Real-Time System (Biorad, Hercules, CA, USA) using the Taqman 5′ nuclease assay PCR method for detection and quantification of bacterial DNA. Primers and probes were targeted against the 16 S rRNA gene for the test group and for the negative control. Taqman reactions contained 12.5 µl Mastermix (Eurogentec, Seraing, Belgium), 4.5 µl sterile H_2_O, 1 ml of each primer and probe, and 5 ml template DNA. Assay conditions for all primer/probe sets consisted of an initial 2 min at 50 °C, followed by a denaturation step at 95 °C for 10 min, followed by 45 cycles of 95 °C for 15 s and 60 °C for 60 seconds. Quantification was based on a plasmid standard curve (Table 4).

**Table 4:** Primers and probes used for the detection and qualification by vitality qPCR.

STRAIN		Primer/Probe (5′-3′)	Fragment length
*P*. *gingivalis*	Forward	GCG CTC AAC GTT CAG CC	68 bp
	Reverse	CAC GAA TTC CGC CTG C	
	Probe	CAC TGA ACT CAA GCC CGG CAG TTT CAA	
*P*. *intermedia*	Forward	CGG TCT GTT AAG CGT GTT GTG	99 bp
	Reverse	CAC CAT GAA TTC CGC ATA CG	
	Probe	TGG CGG ACT TGA GTG CAC GC	
*F*. *nucleatum*	Forward	GGA TTT ATT GGG CGT AAA GC	162 bp
	Reverse	GGC ATT CCT ACA AAT ATC TAC GAA	
	Probe	CTC TAC ACT TGT AGT TCC G	
*A*. *actinomycetemcomitans*	Forward	GAA CCT TAC CTA CTC TTG ACA TCC GAA	80 bp
	Reverse	TGC AGC ACC TGT CTC AAA GC	
	Probe	AGA ACT CAG AGA TGG GTT TGT GCC TTA GGG	

#### Colony counting (CFU/ml)

All samples were also diluted from 10^−1^ to 10^−9^ in PBS and plated by means of a spiral plater on blood agar plates supplemented with 5 mg/ml hemin (Sigma, St. Louis, USA), 1 mg/ml menadione, and 5% sterile horse blood. After 7 days of anaerobic (80% N_2_, 10% H_2_ and 10% CO_2_) or aerobic (5% CO_2_) incubation (depending on the bacteria), the total number of CFU/ml was counted by means of Fiji software (Image Processing and Analysis in Java, 1.8.0_77). Briefly, all images were transformed into a 16-bit-image and a region of interest (ROI) with all colonies was selected. The image was then converted into a binary image. The latter was modified (watershed) to improve the separation of each colony when the colonies were too close. Finally, the count was performed based on the size and the circularity^43^ (Fig. S1). If the number of colonies on an agar plate was above 300, this plate was considered not countable. CFU/ml were calculated according to the dilution point.

#### Gram negative staining

Given the differences in values between the qPCR and the CFU reported in Table 3, a gram negative staining was performed for *A*. *actinomycetemcomitans* (control and ratio 1:1). The samples were analysed with the Axio Imager 2 Microscope (Carl Zeiss MicroImaging GmbH, Germany) with a 40x magnification.

### Ethical Statement

The use of human blood was approved by the ethical committee of the KU Leuven and registered with identifier B322201628215. The procedures were executed according to the Helsinki Declaration and the regulations of the University Hospital, approved by the ethical committee. Informed consent was obtained from all subjects after the purpose of the study had been explained. The subjects were aware that the results would be used in a scientific study.

### Statistical analysis

For all variables, mean values and standard deviations were calculated. The normality of the data was tested with the Kolmogorov-Smirnov test. The normally distributed data were then analysed with Student’s T-test (parametric test) and the 95% confidence interval was computed. For non-parametric data, the Wilcoxon test was used. A p value < 0.05 was considered statistically significant.

## Supplementary information

Supplementary Figure S1

## Data Availability

The datasets generated during and/or analysed during the current study are available from the corresponding author on reasonable request.
