# Supplementary material for: Antimicrobial capacity of Leucocyte-and Platelet Rich Fibrin against periodontal pathogens
Source: Sci Rep. 2019 Jun 3;9:8188. doi: 10.1038/s41598-019-44755-6 (PMC6547693; doi:10.1038/s41598-019-44755-6)

Antimicrobial capacity of Leucocyte-and Platelet Rich Fibrin against periodontal pathogens

Ana B. Castro*[1], Esteban R. Herrero[1], Vera Slomka[1], Nelson Pinto[2], Wim Teughels[1], Marc Quirynen[1]

[1] Department of Oral Health Sciences, Periodontology, KU Leuven & Dentistry, University Hospitals Leuven, Belgium.

[2] Faculty of Dentistry, Postgraduate Implant Program, University of the Andes, Santiago, Chile.

Figure S1. Colony forming units from *P. gingivalis*. Ratio 1:1 : 150 µl bacteria + 150 µl L-PRF exudate, Ratio 1:2 : 150 µl bacteria + 75 µl L-PRF exudate + 75 µl saline, and Ratio 1:4 : 150 µl bacteria + 37.5 µl L-PRF exudate + 112.5 µl saline. Control+: 150 µl bacteria + 150 µl saline. A: standardized RGB color image; B: binary image.

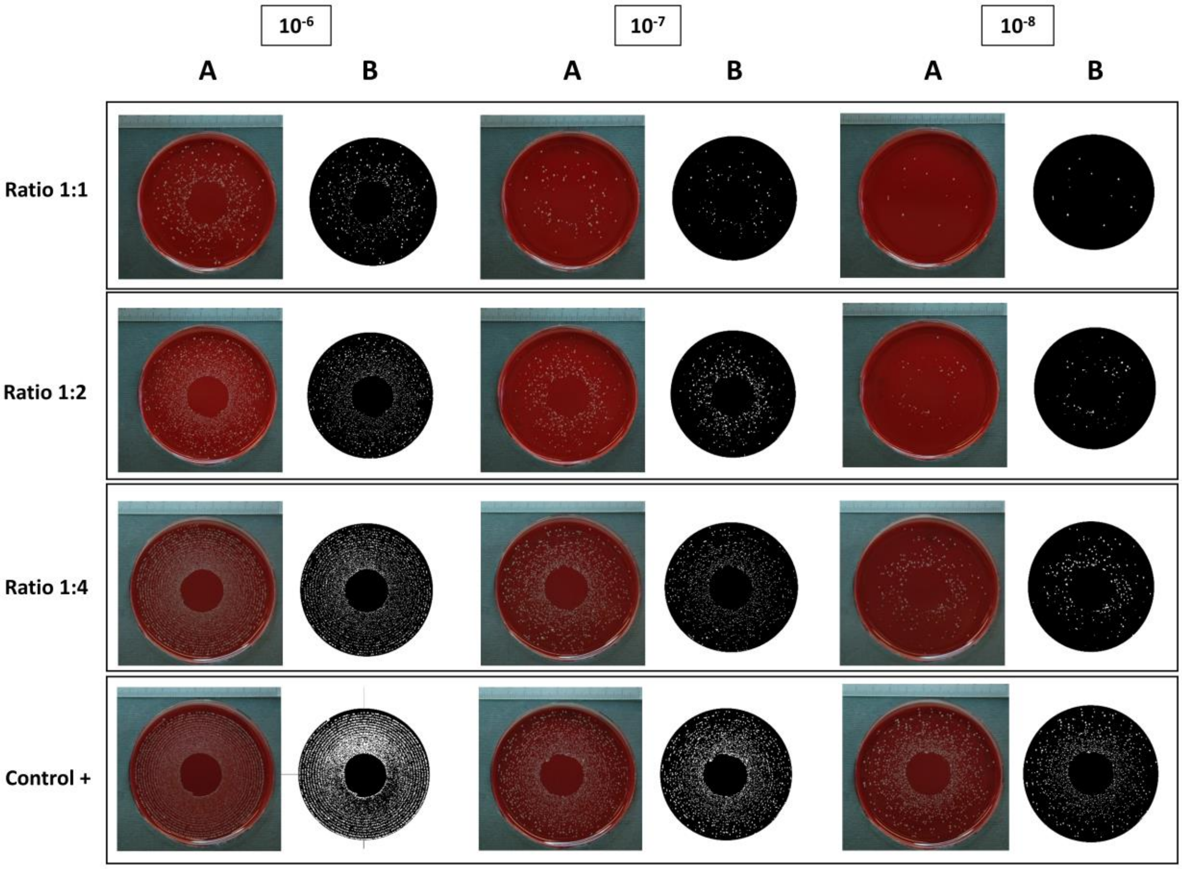

Supplement: Supplementary file 1 — Supplementary Figure S1 [file 41598_2019_44755_MOESM1_ESM.pdf]
